# Predominantly symplastic phloem unloading of photosynthates maintains efficient starch accumulation in the cassava storage roots (*Manihot esculenta* Crantz)

**DOI:** 10.1186/s12870-021-03088-1

**Published:** 2021-07-03

**Authors:** Kun Pan, Cheng Lu, Peixian Nie, Meizhen Hu, Xincheng Zhou, Xin Chen, Wenquan Wang

**Affiliations:** 1 Institute of Tropical Bioscience and Biotechnology, Chinese Academy of Tropical Agriculture Sciences, Haikou, 571101 China; 2 Hainan Medical University, Haikou, 571199 China; 3 Shandong Institute of Pomology, Shandong Academy of Agricultural Sciences, Taian, 271000 Shandong China

**Keywords:** Cassava, Carbohydrates, Storage root, Symplastic unloading, Starch yield

## Abstract

**Background:**

Cassava (*Manihot esculenta* Crantz) efficiently accumulates starch in its storage roots. However, how photosynthates are transported from the leaves to the phloem (especially how they are unloaded into parenchymal cells of storage roots) remains unclear.

**Results:**

Here, we investigated the sucrose unloading pattern and its impact on cassava storage root development using microstructural and physiological analyses, namely, carboxyfluorescein (CF) and C^14^ isotope tracing. The expression profiling of genes involved in symplastic and apoplastic transport was performed, which included enzyme activity, protein gel blot analysis, and transcriptome sequencing analyses. These finding showed that carbohydrates are transported mainly in the form of sucrose, and more than 54.6% was present in the stem phloem. Sucrose was predominantly unloaded symplastically from the phloem into storage roots; in addition, there was a shift from apoplastic to symplastic unloading accompanied by the onset of root swelling. Statistical data on the microstructures indicated an enrichment of plasmodesmata within sieve, companion, and parenchyma cells in the developing storage roots of a cultivar but not in a wild ancestor. Tracing tests with CF verified the existence of a symplastic channel, and [^14^C] Suc demonstrated that sucrose could rapidly diffuse into root parenchyma cells from phloem cells. The relatively high expression of genes encoding sucrose synthase and associated proteins appeared in the middle and late stages of storage roots but not in primary fibrous roots, or secondary fibrous roots. The inverse expression pattern of sucrose transporters, cell wall acid invertase, and soluble acid invertase in these corresponding organs supported the presence of a symplastic sucrose unloading pathway. The transcription profile of genes involved in symplastic unloading and their significantly positive correlation with the starch yield at the population level confirmed that symplastic sucrose transport is vitally important in the development of cassava storage roots.

**Conclusions:**

In this study, we revealed that the cassava storage root phloem sucrose unloading pattern was predominantly a symplastic unloading pattern. This pattern is essential for efficient starch accumulation in high-yielding varieties compared with low-yielding wild ancestors.

**Supplementary Information:**

The online version contains supplementary material available at 10.1186/s12870-021-03088-1.

## Background

Photosynthesis and the transport and accumulation of carbohydrates are the three essential physiological processes of yield formation for any economically important crop [1]. However, the ways in which how photoassimilates are transported from leaf sources to storage sinks is unexpectedly diverse among species. Phloem loading in leaves and unloading at the sink are two restricted steps that play a pivotal role in this process and can significantly affect crop yield and plant productivity [2]. To date, three general sucrose unloading models, namely, symplastic, apoplastic, and mixed models, have been identified in plants. Symplastic phloem unloading, in which sucrose passes through the plasmodesmata between phloem companion cells (CCs) and parenchyma cells (PCs) and into sink tissues is the principal pathway for most plant species [2]. A series of genes related to the formation of plasmodesmata has reported via transcriptomics [1]. Sucrose synthase (SuSy) catalyzes the reversible conversion of sucrose into fructose, and uridine diphosphate glucose acts as a carrier to maintain a physiologically low concentration of sucrose in parenchymal cells. Compared with INV, SuSy is a component of postsymplastic unloading and is more important for starch and protein synthesis [1, 3]. The regulation of symplastic transport mediated by changes in plasmodesmata has been extensively studied in many plant species [4–6].

Apoplastic unloading depends on sucrose transporters (SUTs) to take up sucrose from the phloem to parenchyma cells in storage roots. This process is accompanied by post-unloading invertases. Invertases are promising candidates for mediating the sink strength because they catalyze the irreversible cleavage of sucrose to glucose and fructose. Apoplastic invertase (a cell wall-bound acid invertase), which is associated with apoplastic unloading [1, 7], enables the storage of osmotically active solutes against a concentration gradient [8]. Alternatively, the sucrose in the apoplastic space between cells can be transported via sucrose transporters (SUTs) located on the plasma membrane [9, 10].

The unloading processes can vary on the basis of the sink type, developmental stage, and the function of the genes involved [2]. The transition between two unloading pathways is closely related to the soluble sugar content and invertase activity [11, 12]. Sucrose utilization is initiated by cleavage catalyzed by SuSy and invertase within the organs involved; and this cleavage in turn regulates the unloading rate through two distinct phloem unloading pathways [8, 13]. An inverse correlation between the mRNA expression of soluble acid invertase (SAI)- and SuSy-encoding genes has been found, and the balance between SuSy- and SAI-mediated sucrolytic pathways determines the type of unloading pathway and starch accumulation in potato tubers [14].

An understanding of the cellular routes of phloem unloading is needed to enhance plant yield [15], but developing a direct experimental model for complex sink organs due the substantial technical challenges associated with this process is difficult. To date, mechanistic studies of phloem unloading have focused only on fleshy fruits, such as those of grape [16], Chinese jujube [17], peach [10], blueberry [18], and kiwifruit [19], and few have been dedicated to vegetative storage sinks. Cassava (*Manihot esculenta* Crantz) is a tropical perennial root crop species that is harvested yearly, with an approximately 12-month growth cycle. Cassava ranks as the fifth most important food crop worldwide, and approximately one billion people rely on its starch-rich roots [20]. High-efficiency photosynthesis, tolerance to drought, and extraordinarily high starch accumulation in its storage roots are essential biological characteristics of cassava [21]. Moreover, cassava has three root types: primary and secondary fibrous roots and storage roots [22]. Storage roots develop from primary fibrous roots (PFRs) by following a distinct pattern of secondary growth, and starch can constitute up to 85% of the dry weight of storage roots [23]. However, how such a large amount of carbohydrates are loaded into the phloem in the leaves and unloaded into parenchymal cells in the storage roots is poorly understood.

In this study, we determined the general transport models of carbohydrates in cassava, clarified that sucrose is the major transported component, discovered a predominantly symplastic phloem-unloading system in the storage roots, and found that there was a shift from apoplastic to symplastic transport at the beginning of the fibrous root swelling stage, which is essential for highly efficient starch accumulation in cassava. These findings are based on structural, physiological, and biochemical experiments on developing storage roots, which included measurements of the activity of enzymes and the expression of mRNAs of genes involved in transport and post-transport events.

## Results

### Sucrose is the primary component of Photosynthates transported in the phloem of cassava

**Fig. 1:**
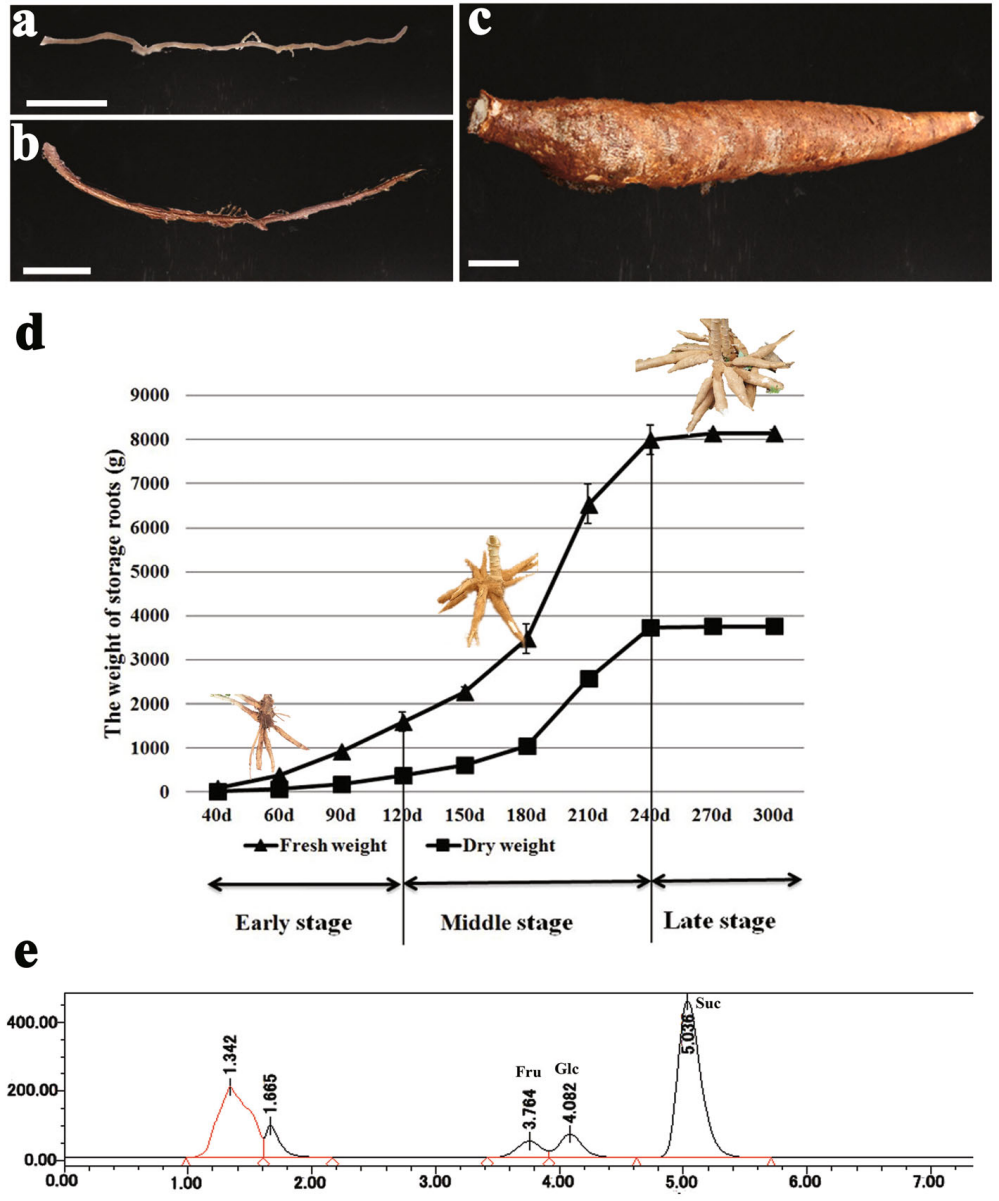
The morphological characteristic, biomass accumulation, substances transported in cassava root. **a** Primary fibrous root. **b** Secondary fibrous root. **c** Storage root. **d** The curves of biomass accumulation of cassava storage root in the three growth stages. The values of the fresh and dry weight are the means of three replicates ±SD. **e** HPLC–ELSD chromatograms of the cassava phloem exudates. The yield curve in **d** was performed by Micro software Excel 2016. All of them were with cropped edges and adjusted position using Adobe Photoshop CS6.0 software. Bars in **a–c,** 1 cm

Cassava plants were potted in a greenhouse. The biomass accumulation curve across three growth stages, the anatomical structure of the storage roots, the results of the phloem sap collection and the phloem composition are shown in Fig. 1. Phloem sap was collected from the stem of plants during development of the storage roots (approximately 60 days after plant emergence (DAPE)). High performance liquid chromatography (HPLC) based examination showed that sucrose had the largest peak, accounting for approximately 79% of the phloem sap, followed by glucose and fructose (Fig. 1e). The concentrations of sucrose, glucose, and fructose in the phloem sap constituted 54.6, 22.7, and 22.5%, respectively (Table 1). These results indicate that sucrose is the main form of carbohydrate transported from leaves to the belowground cassava storage roots.

**Table 1:** The sugar components of the phloem sap of cultivated Arg7 cassava

sugars	samples	Mean Content of sugars (mg·ml^-1)^	Percentage (%)
Sucrose	Arg7	5.687 ± 0.011	54.6
Glucose	Arg7	2.370 ± 0.004	22.7
Fructose	Arg7	2.342 ± 0.025	22.5

### The SE-CC complex is Symplastically connected to surrounding PCs through Plasmodesmata in storage roots

**Fig. 2:**
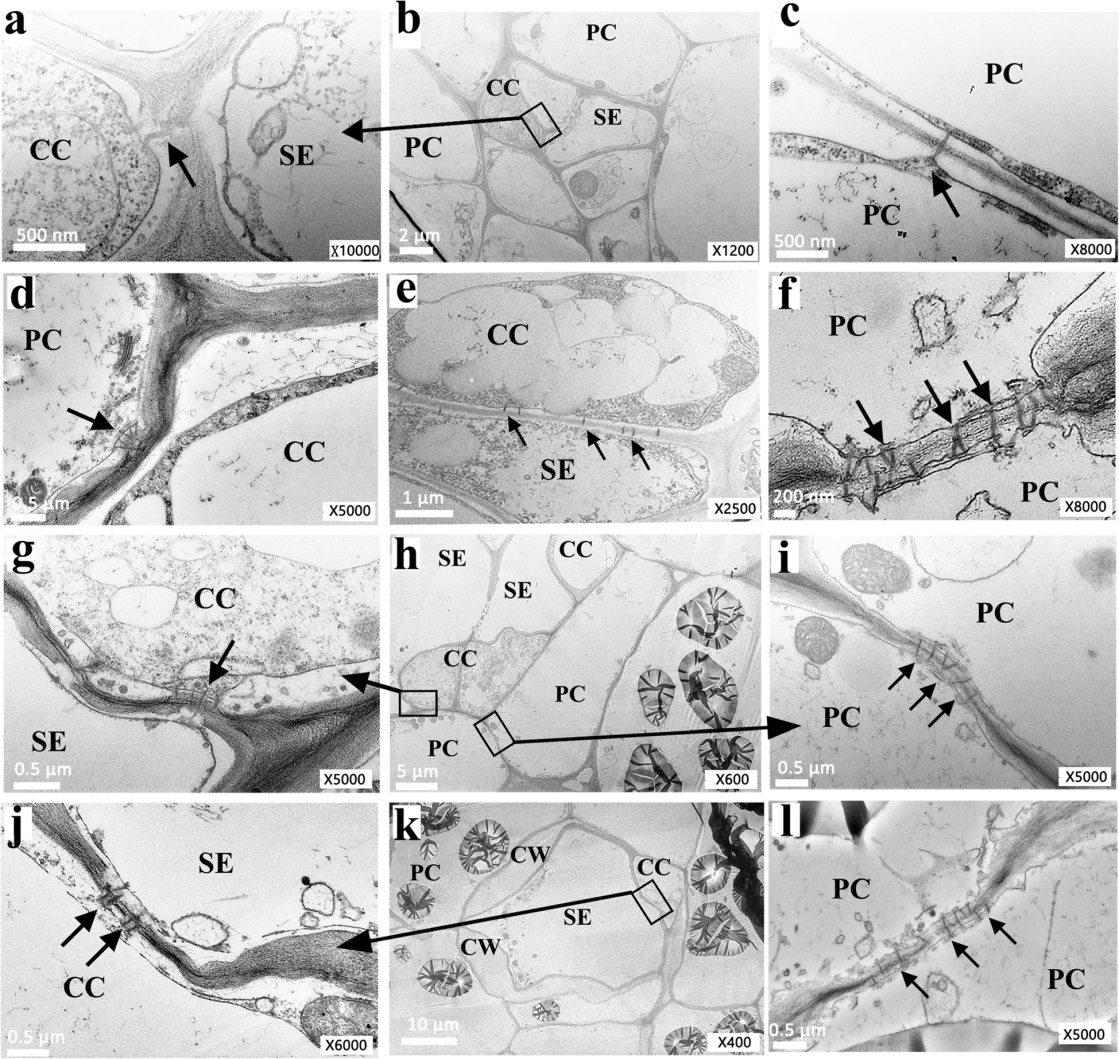
Ultrastructure of phloem cells in storage root of cassava showing highly dense plasmodesmata. **a–c, d–f, g–i, j–l** Phloem cells in the primary fibrous root and storage root at the early, middle, and late stages, respectively. **a, g, j** a partial enlargement of **b, h and k** respectively. **c, f, i, l** Arrows indicated plasmodesmata between two PCs in the primary fibrous root and storage root at the early, middle, and late stages, respectively. **d, e** Plasmodesmata between a PC and adjacent CC cell, between SE and CC in early storage roots respectively. **h, k** A larger scope of the transverse section of the phloem cell group in the storage root at the middle and late stages. CW in **k** indicates a thickened cell wall. All of them were with cropped edges and adjusted position using Adobe Photoshop CS6.0 software. Abbreviations, SE, sieve element; CC, companion cell; PC, parenchyma cell; CW, cell wall

The microstructures of the three kinds of cassava roots (Fig. 1a–c) were investigated in detail, including the control of fibrous roots (see Additional file 1: Fig. S1). According to the biomass (fresh and dry weights) accumulation curve of the storage roots, we classified the developing storage roots into three stages: the early stage (60 DAPE, initial storage roots), middle stage (180 DAPE, fast growth of storage roots), and late stage (270 DAPE, mature storage roots) (Fig. 1d). Our observation particularly focused on the plasmodesmata among phloem sieve elements (SEs), CCs, and PCs (Fig. 2). Plasmodesmata were rarely observed in fibrous roots (Fig. 2a–c), particularly between the SE–CC complex and PCs (Table 2). However, a mass of plasmodesmata was observed between the SEs and CCs (Fig. 2e, g, j), the SE–CC complex and PCs (Fig. 2d), and between PCs (Fig. 2f, i, l) in storage roots during the three developmental stages. In the expanding SEs, one SE was always accompanied by two middle- or late-stage CCs (Fig. 2h, k), and the majority of the plasmodesmata were unilateral in the early stage (Fig. 2d, e, f). However, a number of simple (nonunilateral) plasmodesmata were appeared in the middle and late stages (Fig. 2g, j, i, l). Thick-walled SEs sometimes emerged during the late stage of development (Fig. 2k) in the pore-plasmodesma (PD) units (PPUs) connected to CCs or PCs (see Additional file 2: Fig. S2). The plasmodesmal density in cross-sections containing several cell types was calculated (Table 2). PD numbers among the three types of storage roots cells tended to increase in the three developmental stages but were very scarce in the fibrous roots, especially in the PFRs, as the PDs were mostly present at the interface of the SE–CC complex. The number of PDs increased at the interface between two adjacent PCs compared to those between other interfaces, even in the secondary fibrous roots (SFRs), with limited starch storage capacity. The ultrastructure of the phloem and the distribution of plasmodesmata between the SE–CC, SE–CC complex, PCs, and adjacent PCs in the developing storage roots indicate the channel possibilities of the symplastic transport and communication characteristics of cassava.

**Table 2:** Plasmodesmata density in the cell walls of SE-CC and PC cells in the late storage and fibrous roots of cultivated Arg7 cassava

Plasmodesmata density (Number per μm)			
SE-CC		SE-CC/PC	PC/PC
Primary fibrous root	0.013 ± 0.021	0.007 ± 0.019	0.030 ± 0.032
Secondary fibrous root	0.008 ± 0.007	0.001 ± 0.001	0.049 ± 0.022
Elongation stage of tuberous root	0.033 ± 0.026	0.022 ± 0.015	0.095 ± 0.023
Middle stage of storage root	0.040 ± 0.024	0.027 ± 0.018	0.147 ± 0.035
Later stage of storage root	0.032 ± 0.025	0.021 ± 0.021	0.100 ± 0.065

Plasmodesmata were counted on five scopes that were selected from six ultrathin sections for each sample, and the value above are the means ±SE (individuals). Abbreviations: SE, sieve element; CC, companion cell; PC, parenchyma cell

### Rapid lateral diffusion of Carboxyfluorescein (CF) strongly support the predominance of Symplastic phloem unloading in developing storage roots

**Fig. 3:**
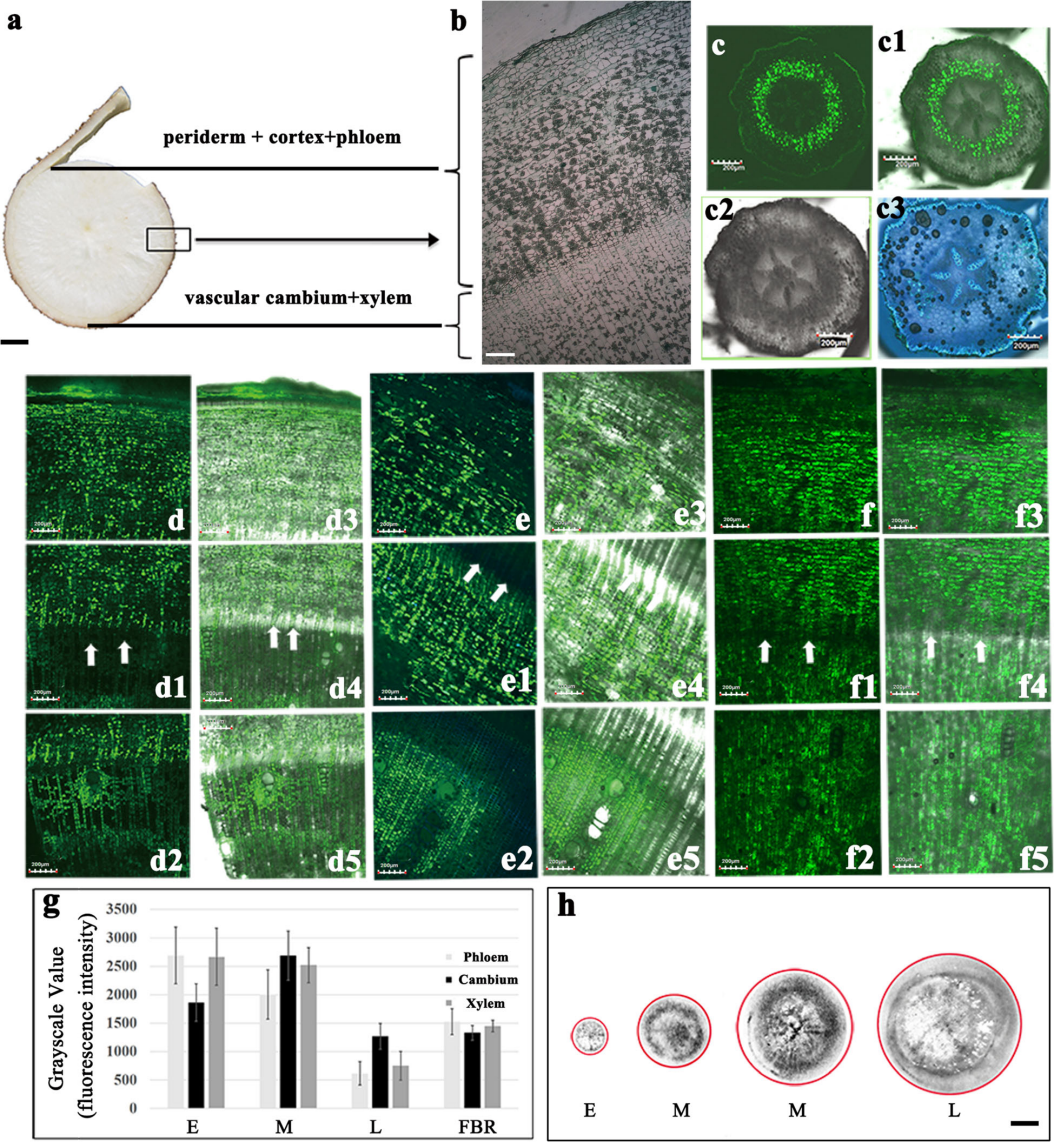
Carboxyfluorescein (CF) and [^14^C] Suc tracing support a predominantly symplasmic phloem unloading pathway in the storage root of cassava. **a, b** Transverse section anatomy of the storage root. **c–f5** CLSM imaging of CF unloading during cassava root development. **c–c3** Primary fibrous root. **c2** Bright–field microscopy. **c3** 405nm excitation wave and bright–field microscopy. **d–d5** Early stage storage root. **e–e5** Middle stage storage root. **f–f5** Late stage storage root. **c1, d3–d5, e3–e5, f3–f5** Bright–fiield and 488 nm excitation wave microscopy. **g** CF fluorescence intensity of different root develop–mental stages. **h** The difference in the distribution density of [^14^C] Suc in cross sections of developmental roots at the three stages. The arrows indicate the vascular cambium. All of them with cropped edges and adjusted position using Adobe Photoshop CS6.0 software. Abbreviations, E, Early stage of storage root; M, Middle stage of storage root; L, Late stage of storage root; FBR, Primary fibrous root. Bar = 5 cm in **a**; Bar = 1 μm in **b**; Bar = 200 nm in **c-f5**; Bar = 1 cm in **h**

Upon entering the cells, membrane-permeable nonfluorescent 6(5)-carboxyfluorescein diacetate (CFDA) is broken down to 6(5) carboxyfluorescein (CF), which is a membrane-impermeable fluorescent dye. CF is often used as a fluorescent marker of phloem transport and symplastic phloem unloading [11]. In this study, it was injected into the stem near the base of cassava plants at the early, middle, and late developmental stages. After 72 h, the CF green fluorescent signals were detected immediately in the roots at the three stages via microsections (Fig. 3a, b). CF molecules were extensively distributed throughout the phloem (SPH) and xylem (SXY) regions of the storage roots at the three developmental stages (Fig. 3d–f5), and the highest density of CF predominantly appeared at the early and middle stages (Fig. 3d–e5, g). In contrast, the CFs were restrictedly released along the cortex but were not visible in the primary phloem (PPH) or primary xylem (PXY) in the PFRs (Fig. 3c, c1). There was no distribution of CF green fluorescence in the PFRs under bright-field microscopy (Fig. 3c2) or excitation at 405 nm (Fig. 3c3). This CF tracing experiment clearly illustrated that there are channels allowing CFs with molecules similar to sucrose to move through the cell membrane driving fast spreading among the parenchyma cells of the xylem in the storage roots, especially at the middle developmental stage. We further utilized [^14^C] Suc tracing to inspect the diffusion strength difference of the sucrose molecules marked across the three developmental stages. In this stage of the experiment, [^14^C] Suc was fed to the stalk at the base of the plants, and after 72 h, we investigated its distribution and density in the roots at the three stages (Fig. 3h). The ^14^C accumulated immediately in the phloem and xylem tissue of the developing storage roots. The [^14^C] Suc signal was stronger in the storage roots at the middle stage than at the late stage, and the signal in the early stage was very weak. This ^14^C unloading trend in the roots at the three key developmental stages is in agreement with the demands of the growth and biomass accumulation characteristics of cassava plants (Fig. 1d). The fast unloading and dynamic change in sucrose traced by [^14^C] Suc proved that the vascular bundle has carbohydrates transportation function, and the existence of symplastic space identified by CF transport strongly supports the idea that a symplastic phloem unloading pathway exists in cassava storage roots.

### Differential expression level of genes involved in Postphloem transport and the relevant activity of their encoded enzymes further support Symplastic unloading in cassava storage roots

Sucrose molecules are broken down immediately by SuSy following symplastic unloading or by INV in the case of apoplastic unloading. The sucrose transporter SUT is the main regulator of apoplastic sucrose transport [10, 24]. We compared the expression levels of genes encoding SUTs, SuSy, and cell wall acid invertase (CWI) and their enzyme levels in the leaves, different root types, and storage roots of plants at the three developmental stages.

**Fig. 4:**
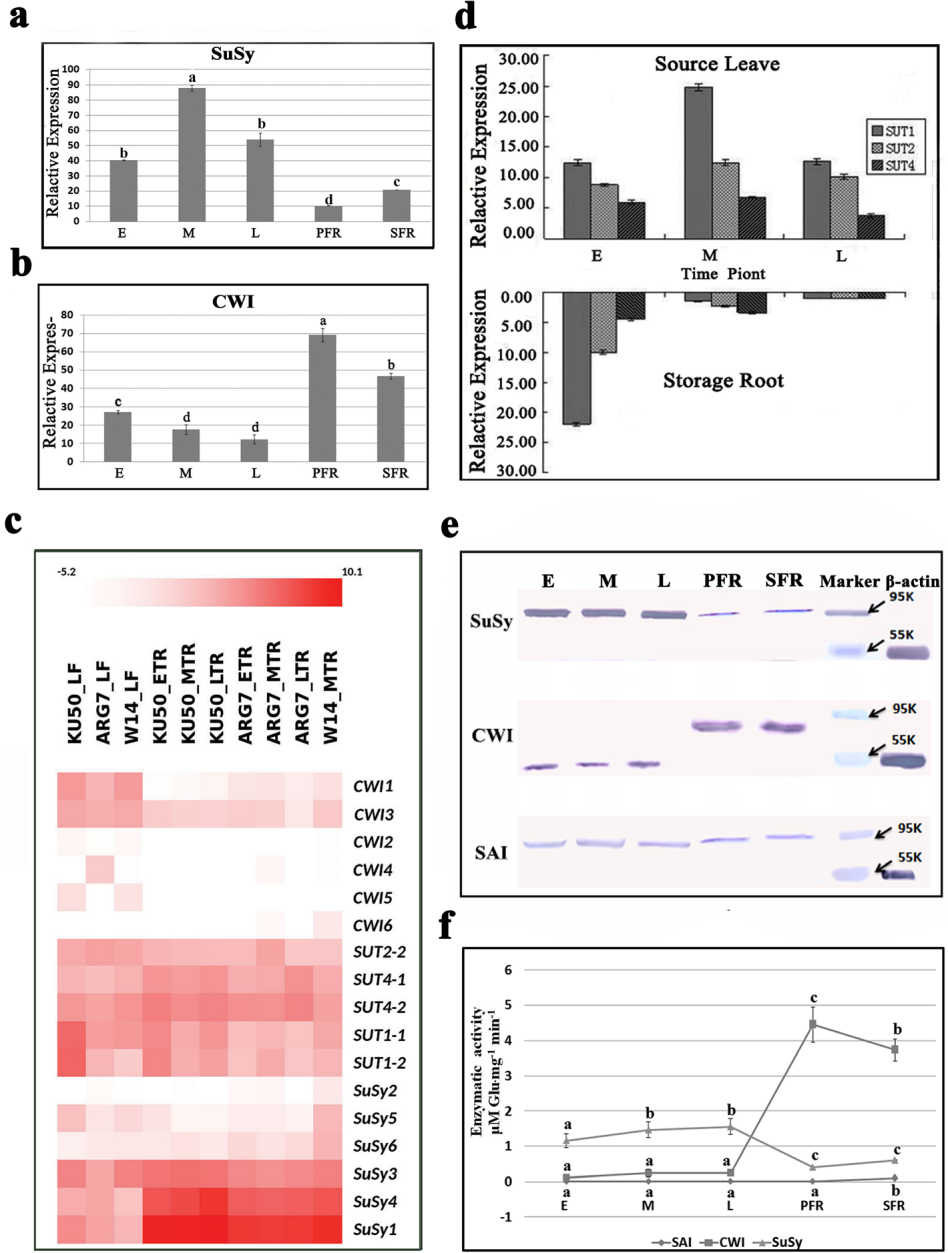
The expression and enzymatic activities of genes involved in post phloem unloading in the storage root of cassava. **a, b** Relative expression difference of CWI and SuSy measured by Q–PCR. **c** Heat map comparisons of differential gene expression of CWIs, SuSys and SUTs. **d** The expression profiling of SUTs (SUT1, SUT2 and SUT4) in leaves and storage roots measured by Q–PCR. **e** The expressed protein levels of SuSy, CWI and SAI assessed by immunoblotting. **f** Enzymatic activities of SuSy (triangles), CWI (squares) and SAI (circles). The column chart in **a, b, d** and the curve chart in **f** were performed by Micro software Excel 2016. All of them were with cropped edges and adjusted position using Adobe Photoshop CS6.0 software. E, Early stage of storage root; M, Middle stage of storage root; L, Late stage of storage root; PFR, Primary fibrous root and SFR, Secondary fibrous root; a, b and c denote significantly different levels at *P* < 0.05

SuSy was highly expressed in the storage roots of plants at the three developmental stages but less expressed in the PFRs and SFRs (Fig. 4a), whereas CWI expression was higher in the PFRs and SFRs than in the storage roots of plants at the three developmental stages (Fig. 4b). SuSy-encoding genes, including SuSy1, SuSy3, and SuSy4, were more highly expressed than CWI- and SUT-encoding genes in the storage roots of plants at the three developmental stages (Fig. 4c). Even though SUTs were expressed in the storage roots (Fig. 4c), SUT members such as SUT1, SUT2, and SUT4 were much more highly expressed in the leaves, except in the fibrous roots of plants at the early developmental stage (Fig. 4d). The protein expression levels of SuSy, CWI, and SAI in the different roots and at the key developmental stages of the storage roots were measured via protein gel blot analysis (Fig. 4e) (the full-length gels blots see Additional file 3: Fig. S3). The expressed protein level of SuSy in storage roots during the early, middle, and late stages was significantly higher than thosein the PFRs and SFRs, whereas the CWI protein level was markedly lower in the storage roots at the three stages than in the PFRs and SFRs. The SAI protein quantity was consistently low, with no apparent change between the tested root types. The enzyme activities were very consistent with their protein levels, and CWI was significantly active in the PFRs and SFRs was but later replaced by SuSy in the storage roots (Fig. 4f). The quantified results above show that the high expression of SuSy and low expression of SUTs and CWI in the developing storage roots as well as the opposite expression in the primary fibrous roots and secondary fibrous roots are all indicators of a sucrose symplastic unloading pathway in cassava storage roots, which developed from primary fibrous roots at the early stage of cassava plant growth.

**Fig. 5:**
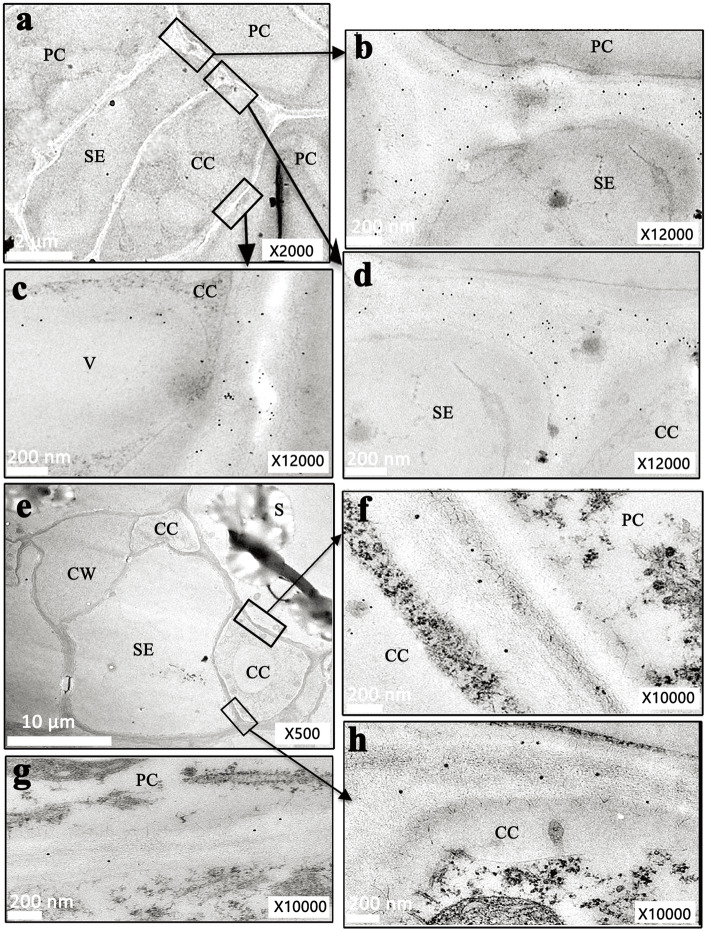
Subcellular location of CWI presented its enrichment in early fibrous roots using immunogold particle labeling. **a–d** Immunogold labeling of CWI in primary fibrous roots with an antibody directed against cassava acid invertases. **e–h** Immunogold labeling of CWI in storage roots with an antibody directed against cassava acid invertases. All of them were with cropped edges and adjusted position using Adobe Photoshop CS6.0 software

Immunogold labeling assays revealed that CWI molecules, which are biomarkers of apoplastic transport of sucrose, were enriched in the early fibrous roots but not in the developing storage roots. The labeled CWI proteins were visible predominantly on the cell walls between the SEs and PCs, CCs and PCs, and SEs and CCs (Fig. 5a–d) of the PFRs. However, very few CWI proteins were found in the cell wall spaces between CCs and PCs, SEs and CCs, and between PCs and PCs (Fig. 5e–h) in the storage roots at the middle or late developmental stages. No CWI molecules were observed in any control without antiserum or preimmune serum (data not shown), indicating that the antiserum was highly specific and that nonspecific labeling was negligible. In summary, the results also suggested that there is a predominantly symplastic phloem unloading pathway in developing cassava storage roots.

## Discussion

Cassava (*Manihot esculenta* Crantz), which is known as one of the most important tuber and root crop species, is a typical tropical crop species with a high potential for starch accumulation. It has a high photosynthesis capacity with photosynthesis characteristics between those of C3 and C4 plants, which indicates that a large amount of carbohydrates are transported from sources to the belowground sink tissues. Accession W14 of the wild cassava ancestor species, which has very low starch yield, presented very low numbers of plasmodesmata in its storage roots (unpublished data). Patrick and Offler [25] found that predominantly symplastic unloading was associated with a high transport capacity and low resistance. Plasmodesmata provide symplastic continuity between most adjacent cells in higher plants and thus substantially contribute to phloem unloading into sink tissues [26]. Our research showed that the number of plasmodesmata increased with the expansion of storage roots, unilateral plasmodesmata were mainly found between SEs and CCs, and simple plasmodesmata were found between PCs and accounted for the majority of phloem unloading cells. Simple plasmodesmata are “open”, and unilateral plasmodesmata are associated with low conductivity [5, 27]. The cell walls of cassava storage roots thickened during the late stage (Fig. 2k), but minimal changes were observed in the amount of plasmodesmata during storage. This was different from the case in apple [28] and jujube [17], wherein the plasmodesmata were blocked during the shift of the unloading pathway.

Storage roots undoubtedly develop from special fibrous roots in cassava under some conditions [22]. This induces a change in the belowground roots to becoming nutrient sinks along with intense changes in structure. The energy cost of apoplastic unloading is replaced by the more effective cost of symplastic unloading. Sucrose is the dominant photosynthate in cassava phloem exudates, and carbohydrates (mainly starch) accumulate rapidly during the early and middle developmental stages of cassava storage roots, while they accumulate slowly during the late developmental stage. This is different from the mechanism in fleshy fruits, wherein the accumulation of a high concentration of soluble sugars relies on apoplastic phloem unloading [10, 16, 29]. In other tuber or root crop species, such as potato [11], carrot [30], and radish [31], switches from apoplastic unloading to symplastic unloading occur during the development of the swelling roots. Thus, a similar switch occurs in cassava from fibrous roots to storage roots, and the switch node may be a critical developmental checkpoint for cassava storage roots.

In actuality, in terms of immediately breaking down or removing sucrose from intracellular space, this postunloading event is extremely important for the lack of a concentration gradient involved in the symplastic transport of sucrose. The expression of SuSy, which is responsible for sucrose conversion into glucose and fructose, is significantly correlated with symplastic phloem unloading and driving sucrose conversion into starch, cellulose, and lignin, which then accumulate in sink organs [13, 15, 32]. SuSy is considered a biomarker of storage sink capacity [33–35]. SuSy, but not CWI or SAI, was identified at the expression and protein levels and was maintained at high concentrations in the storage roots at the early, middle, and late developmental stages of cassava roots. This feature was also found in kiwifruit and apple [19, 36]. In contrast, SUTs, which are key players in the apoplastic transport of sucrose, were expressed at much higher levels in the leaves at all three developmental stages and were expressed more in the fibrous roots than in the storage roots of cassava at the middle and late stages. There appeared to be a predominant apoplastic loading mechanism in the leaves of cassava. This had already been investigated by us in a doctoral thesis. For sucrose unloading in roots, SUT-dependent positive unloading plays a much greater role in the primary fibrous roots and secondary fibrous roots than in the storage roots, which was different from the role of sucrose unloading in the fruits of grape or jujube [16, 17]. CWI, which catalyzes the irreversible conversion of sucrose into glucose and fructose, is associated with phloem apoplastic transport. CWI is strictly expressed in dormant apical buds, but it has been isolated from the symplasts in potato tubers [11]. In cassava, we found that CWI was obviously less expressed in the storage roots than in the fibrous roots, and plasmodesma formation occurred in the primary structure of the roots. Therefore, our results inferred that the cambium of the primary storage roots may also be symplastically isolated in cassava and functionally connected to the symplast in the parenchyma at the onset of differentiation. Afterward, the number of plasmodesmata connected to the pericycle cells increases. Reports on *Arabidopsis* stated that CF and free green fluorescent protein (GFP) could be symplastically unloaded from the phloem into cells of the root cortex and epidermis [37, 38]. We also found that CF was restricted to the endodermis in primary fibrous roots (Fig. 3j). SAI is another sucrose-catabolizing enzyme that is localized in the vacuole and participates in the breakdown of sucrose inside cells, especially in soluble sugar-accumulating sinks such as *Camellia oleifera* fruits [12]. In this study, SAI exhibited a lower level and minimal changes in the cassava roots at different developmental stages. The unloading pathway was linked to concentration differences in total soluble sugars between source and sink cells [15]. As in cassava, high levels of soluble sugars could increase turgor pressure, and the steepness of the turgor gradient is the driving force of symplastic phloem unloading by means of bulk flow through plasmodesmata [2, 39]. This phenomenon is generally ensured by the subcellular compartmentalization of sucrose in sink cells and the utilization of imported metabolites for the production of high-molecular-weight storage compounds or for cell growth [1, 4]. The relatively strong correlations between the expression level of genes involved in the symplastic unloading and the dry matter yield of cassava cultivars confirmed that this symplastic unloading system, which evolved in storage roots, is essential for the high potential starch accumulation in cassava.

## Conclusions

**Fig. 6:**
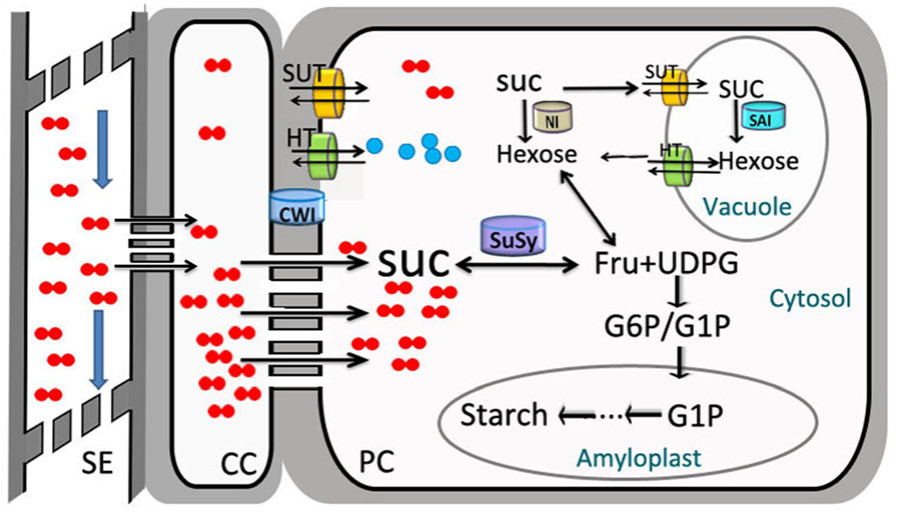
A model of sucrose symplastic phloem unloading in the storage root of cassava. Long–distance transported SUC in the SE enters the CC and PC predominantly through plasmodesmata, with the exception of a few SUCs that are transported by SUTs. These SUC molecules are primarily catabolized by SuSy into fructose and nucleoside diphosphate–glucose (UDPG), which enter starch synthesis in the amyloplast. Occasionally, SUC can be stored temporarily in vacuoles by SUT or HT in a hexose form. This structured energy-saving model accounts for the highly efficient starch accumulation observed in cassava storage roots. This model diagram was performed by Micro software Powerpoint 2016

Based on the results presented above, we summarized our hypothesis for the sucrose symplastic unloading model in cassava, as shown in Fig. 6:

1. There were increasing numbers of plasmodesmata between the SE–CC and PCs of developing storage roots of cultivated cassava. This allows sucrose, which is the main form of photosynthates, to be efficiently unloaded into parenchyma cells in the storage roots.
2. This symplastic unloading process causes a change in primary fibrous roots, which storage roots, and there is a shift from apoplastic unloading to symplastic phloem unloading following the formation of plasmodesmata.
3. Postphloem unloading events and increased SuSy activity (but not CWI or SAI activity) are responsible for the sucrose degradation and biosynthesis of starch in cassava.

## Methods

### Plant materials

The cassava (*Manihot esculenta* Crantz) cultivars Arg7 and Ku50 and cassava (*Manihot esculenta ssp. flabellifolia*) accession W14, which is a wild ancestor of cassava, were used in this study. W14 was donated by CIAT, Ku50 originated from Argentina, and Arg7 was provided by the Royal Agricultural University of Thailand. All materials were introduced and identified by the Tropical Crop Genetic Resources Institute of the Chinese Academy of Tropical Agriculture Sciences (CATAS), and Arg7, Ku50, and W14 were deposited as MS000581, MS000124, and MS000580, in the resource beds of the Tropical Crop Genetic Resources Institute, CATAS.

Arg7 and Ku50 are cultivars with high-yielding storage roots containing approximately 30% starch contents. Compared with Arg7, W14 presents two- to five-fold lower storage root yields and less than a 5% starch content in its storage roots. Comparisons of the biological indexes between cultivars and the wild ancestor were performed to determine the underlying features of the modern cultivar. Hundreds plants were planted in a greenhouse in early March 2015 for use in the experiments. With respect to the biomass, microstructure, and sampling performed for the different experiments, treatments were applied at the early stage (60 DAPE), at which point the cassava storage roots were forming and elongating; at the middle stage (180 DAPE), at which point the storage roots were expanding and rapidly accumulating starch; and at the late stage (270 DAPE), at which point the storage roots were near their maximum weight or contained the highest density of starch. All original data were collected from three plants, with the average value taken for each genotype.

### CF and [^14^C] Suc tracing

CFDA labeling was performed as described previously [9, 16]. The CFDA solution was introduced into the stem near the base of the cassava plants. A cotton thread was placed in a tube at one end, with the other end passing through the phloem zone of the stem base of the storage roots. This method was selected for its high speed of transport and reliability. After 40 min, the plants were labeled with approximately 500 μl of 1 mg·ml^− 1^ CFDA aqueous solution (prepared from a stock solution in acetone). The tubes were wrapped in aluminum foil to avoid the loss of dye and fluorescence quenching under sunlight, and the CF was allowed to translocate for 72 h (which was selected after a time gradient experiment). Approximately 1000–2000 μl of 1 mg·ml^− 1^ CFDA aqueous solution was used for the developing storage roots. The belowground sink tissues were subsequently sectioned and examined for CF fluorescence using confocal laser scanning microscopy (CLSM) (FV1000, Olympus, Japan). The samples were scanned using an excitation wavelength of 350–600 nm, and the strongest CF fluorescence peak was observed at 488 nm. 35.0% laser transmissivity was selected uniformly, and the image size was 800*800 pixel.

The strongest autofluorescence of cassava roots was detected at 405 nm. We scanned every section using 405 nm and 488 nm excitation wavelengths to determine the distribution of autofluorescence and the green fluorescence of CF.

Sucrose was labeled with the ^14^C isotope under experimental conditions. The resulting [^14^C] Suc was injected into the upper stem according to the same method used for CFDA. After 48 and 72 h, the storage roots were sampled, and paraffin sections were made. The manually cut made sections were gently compressed and autoradiographed in cassettes using a Kodak XBT-1 instrument at 4 °C for 30 d. The distribution of [^14^C] Suc in the storage roots was detected by [^14^C]-autoradiography, and then were scaned by a printer (E408, Canon, Japan) directly.

### Ultrastructural observations and measurement of Plasmodesmal density

Ultrathin sections were prepared as follows. The storage roots were cut into small cubes (approximately 2 mm^3^) that were immediately fixed with 4% (v/v) glutaraldehyde in 100 mM precooled phosphate buffer (pH 7.4) for 4 h at 4 °C. Penetration of the glutaraldehyde buffer was performed manually using a syringe. After an extensive rinse with the precooled phosphate buffer (pH 7.4), the tissue cubes were post-fixed in 1% (w/v) OsO_4_ for 1–2 h and shaken several times during that time period. Following another extensive rinse with the same buffer, then dehydrated through a graded ethanol series (50–100%). Propylene oxide was used to displace the spur epoxy resin, which was then infiltrated for 24 h at room temperature. Polymerization was conducted at 70 °C for 8 h. These sections (approximately 80 nm in thickness) were mounted on 100-mesh copper grids or were nickel coated with 0.25% Formvar film for ultrastructural observations using a TEM (JEM 2100, JEOL, Japan).

The plasmodesmal density was measured as described in [40] and in a previous study [17]. Five serial sections of two orientations (transverse and longitudinal) of ultrathin sections were prepared from Spurr-infiltrated samples. Each group comprised sections located approximately 20 μm apart. From each group, six ultrathin sections were selected at random and placed on copper grids (100-mesh). Samples consisting of phloem and surrounding PCs were selected from each ultrathin section, and five of them were observed via transmission electron microscopy (TEM) (JEM 2100, JEOL, Japan). All cell interfaces (i.e., between SEs and CCs, SEs and PCs, CCs and PCs, and PCs and PCs, with PCs including both phloem parenchyma and xylem storage parenchyma cells) in each selected field were used to count the plasmodesmata. The number of plasmodesmata/interface length is referred to as the plasmodesmal density (No. plasmodesmata·μm^− 1^), and half plasmodesmata were counted as one.

### Histochemical analysis

Antibodies against CWI, SAI, and SuSy were generated against polypeptides by ComWin Biotech Co., Ltd. (China). The gene sequences of CWI were obtained from Phytozome V12.1 (http://www.Phytozome.com), and we selected three isoforms of this enzyme that were expressed in cassava roots (at three different developmental stages): CWI-1, CWI-2, and CWI-5. The first two isoforms with the same amino acids have the partial sequence QPYRTSYHFQPPK, whereas the last isoform has the specific sequence DPKQRQVQNYAVPK. The isoform-specific partial amino acid sequence of SAI was QKGSEQTFPSRE. This sequence was generously provided by Zhang Peng (Institute of Plant Physiology, Shanghai Institutes for Biological Sciences, Chinese Academy of Sciences) and was proven to be highly and specifically expressed in cassava roots. The sequence of SuSy, which was also highly expressed in cassava roots, was generously provided by Dr. Luiz (EMBRAPA Genetic Resources and Biotechnology, Brasilia, DF, Brazil). Its GenBank number is AAV74405.1, and the specific sequence of this isoform is RRKESKDLEEXA (basic information about these genes is shown in Table 3).

**Table 3:** Basic information on the cassava genes of sucrose synthase, cell wall invertase and sucrose transporters

Gene	JGI ID v6.1	Location	(bp)	CDS (bp)	Protein (aa)
*SuSy1*	Manes.03G044400	Chr03:3583270.3588587 reverse	2752	2421	806
*SuSy2*	Manes.03G198900	Chr03:28057541.28064463 reverse	2598*	2241	746
*SuSy3*	Manes.01G221900	Chr01:30850003.30857026 reverse	2867	2436	811
*SuSy4*	Manes.16G090600	Chr16:24754130.24759053 forward	2750	2421	806
*SuSy5*	Manes.02G081500	Chr02:6080529.6085146 reverse	2881	2526	841
*SuSy6*	Manes.14G107800	Chr14:8748090.8752577 forward	3026	2739	912
*SuSy7*	Manes.01G123800	Chr01:24140021.24144607 reverse	2783	2526	841
*CWI1*	Manes.03G049200.1	Chr03:4223184.4226988 forward	2401	1779	592
*CWI2*	Manes.08G027200.1	Chr08:2431570.2433947 forward	1851	1719	572
*CWI3*	Manes.11G025400.1	Chr11:2236114.2240122 reverse	2079	1767	588
*CWI4*	Manes.04G140500.1	Chr04:26727894.26729547 forward	1369	1278	425
*CWI5*	Manes.008G027200.1	Chr08:2431570.2433947 forward	1851	1719	572
*CWI6*	Manes.0009G053500.1	Chr09:7064243.7068843 reverse	1719	1719	572
*SUT1–1*	Manes.18G099400.1	Chr18:8603890.8607266 forward	2193	1785	594
*SUT1–2*	Manes.02G190300.1	Chr02:15510673..15513298 forward	2189	1542	513
*SUT4–1*	Manes.18G054200.1	Chr18:4548075.4559586 forward	3720	1497	498
*SUT4–2*	Manes.05G186600.1	Chr05:25724747.25733136 forward	3144	1491	496
*SUT2–2*	Manes.05G099000.1	Chr05:8333636.8347861 reverse	3028	1827	608

### Histological structural observations

Sections prepared from paraffin-embedded tissues were used for histological structural observations. Small cubes (2–3 cm^3^) of cassava storage roots were immediately fixed in formalin–acetic acid–alcohol (FAA) for 36 h at room temperature and dehydrated through a series of graded ethanol solutions (80–100%). The subsequent material was processed with *n*-butyl alcohol three times and then embedded in paraffin for 12 h at 60 °C–65 °C. Sections were taken under a 5x lens using a microscope (DM2500, Leica, Germany).

### Immunogold labeling

Immunogold labeling was conducted as described in [16]. Briefly, ultrathin sections were prepared as described above, except that they were fixed with 4% glutaraldehyde. The sections were first incubated with rabbit antiserum specific for SAI, CWI, or SUT prepared as described above and then incubated together with secondary antibodies (goat anti-rabbit IgG antibodies conjugated to 10 nm gold). Finally, the sections were stained with alkaline lead citrate and uranyl acetate, then examined with a TEM (JEM 2100, JEOL, Japan). Two negative controls were designed to verify the specificity and reliability of the immunogold-labeling. In the first negative control, the antiserum was omitted, the nonspecific labeling of the goat anti-rabbit IgG antibody-gold conjugate was tested. In the second negative control, in order to determine the specificity of the antiserum, rabbit antiserum was replaced by rabbit preimmune serum prior to immunogold labeling. We have done at least three control repetitions for each sample.

### Extraction of mRNA, determination of mRNA levels and enzyme activity, and protein gel blotting analysis

Total RNA from cassava roots was extracted using RNAplant plus Reagent (TIANGEN, China). Primers for SuSy, CWI, and SAI were designed based on the sequences described above, and amplicons were detected using a real-time quantitative PCR cycler (Rotor-Gene 6000, QIAGEN, German). β-Actin was used as a reference gene.

Enzyme extraction and assays of SAI or CWI activity were performed as described previously [16]. Buffer A medium: 10 mM MgCl_2_, 150 mM Tris-HCl (pH 8.0), 1 mM benzamidine, 2 mM ethylenediaminetetraacetic acid, 0.1 mM phenylmethyl sulfonyl fluoride, 0.2% (v/v)-mercaptoethanol, 3% (w/v) polyvinylpolypyrrolidone and 10 mM ascorbic acid. Four layers of cheesecloth was used to filter the initial liquid, and then subsequently centrifuged at 16,000 g for 20 min, the supernatant was collected for SAI assays. Precipitated residue was used to prepare CWI, the same buffer without polyvinylpolypyrrolidone was used to rinse the residue until it free of protein. Added 0.5 M NaCl to buffer A, which were used to extract the CWI with gentle shaking for 24 h, then centrifugate it, take the supernatant for the CWI enzyme assays. All extraction processes should be carried out at 4 °C. The SAI activity was assayed using soluble and insoluble fractions as described in [41], each assay consisted of 0.3 ml of 100 mM sodium acetate buffer (pH 4.8), 0.1 ml of 100 mM sucrose, and 0.1 ml of enzyme sample.

Enzyme extractions and assays of SuSy activity were performed as described in [42]. Briefly, frozen root tissues were coped with 5 ml of media containing 50 mM HEPES buffer (pH 7.0), 10 mM 2-mercaptoethanol, 1% polyvinylpyrrolidone, 2% polyvinylpolypyrrolidone, 1 mM EDTA, and 10 mM MgCl_2_. Afterward, 0.1 ml of the desalted extract was added to 0.9 ml of reaction media composed of 25 mM HEPES-NaOH buffer (pH 6.5), 125 mM sucrose, 15 mM MgCl_2_, and 2 mM UDP. The enzyme activity in the direction of sucrose cleavage was assayed at 28 °C in the presence of sucrose. The reducing sugars produced were assayed using the 3,5-dinitrosalicylic acid-base method described in [43].

Proteins were extracted according to the methods of Miron and Schaffer [44]. The extraction buffer consisted of 100 mM Tris-HCl (pH 8.9), 250 mM sucrose, 10 mM MgCl_2_, 5 mM vitamin C, and 3.5% crosslinking polyvinylpyrrolidone. (NH_4_)_2_SO_4_ was used to precipitate CWI, SAI, and SuSy proteins, and protein concentrations were determined using the Bradford [45] method, with bovine serum albumin used as a standard. SDS-PAGE and immunoblotting assays were performed as described by Pan [46], with slight modifications. After polyacrylamide gel electrophoresis transfer, the nitrocellulose membrane was blocked. Then incubated it overnight at 4 °C in antisera specific to SAI (1:4000), CWI (1:2000), and SuSy (1:5000), which were diluted with Tris-buffered saline (TBS; 10 mM Tris-HCl, 150 mM NaCl) + 0.05% Tween 20 + 3% bovine serum albumin (BSA). The membranes were then washed in TBS with Tween 20 (TBST; 10 mM Tris-HCl, 150 mM NaCl + 0.05% Tween 20) three times, incubated for 45 min at room temperature with goat anti-rabbit IgG-alkaline phosphatase conjugate, diluted 1000-fold with TBST_2_ (50 mM Tris-HCl, 150 mM NaCl + 0.1% Tween 20 + 1% BSA), and then treated with secondary antibodies. After being washed three times in TBST_2_ and TBS, the membranes were stained with a BCIP/NBT Kit (ComWin Biotech Co., Ltd., China). β-Actin was used as a reference gene.

### Collection and analysis of phloem exudates

Phloem exudates were collected from the stems of 60-day-old seedlings as described by King and Zeevaart [47]. The stem was cut near the base, washed with ultrapure water, and inserted into a solution of 20 mM EDTA (pH 7.5). The detached plant was kept in darkness at 30 °C and 95% humidity for 1 to 2 h and then transferred to ultrapure water for collection for 4–5 h. Phloem exudates were lyophilized and stored at − 80 °C. The sucrose, glucose, fructose, sorbitol, stachyose, and raffinose contents of the exudates were evaluated using HPLC with an evaporative light-scattering detector (ELSD). The samples were analyzed in 10 μl increments with water amide (xBridge 3.5 μm, 4.6 mm × 150 mm, USA). The mobile phase consisted of 70% (v/v) acetonitrile and 0.1% (v/v) ammonium hydroxide; the flow rate was 1 ml min^− 1^; the temperature was 25 °C; the drift tube temperature was 85 °C; the nitrogen flow rate was 2.0 l min^− 1^; and the gain value was 2.

### Transcriptome sequencing and annotation

Ten RNA libraries generated from the developing leaves and storage roots of Ku50, Arg7, and W14 plants were sequenced using an Illumina High-Seq 2000 system, with approximately 100 bp reads. After preprocessing, the mRNA sequence reads from 10 samples were mapped to the draft genome sequence of AM560–2. Approximately 20,000 unigenes were annotated. The genes involved in the development of all RNA-seq reads of the 10 samples were uploaded to the NCBI SRA under the following accession numbers: SRX551093, SRX553797, SRX553799, SRX553800, SRX553801, SRX553802, SRX553803, SRX553804, SRX553805, and SRX553807. Cluster 3.0 software (http://bonsai.hgc.jp/~mdehoon/software/cluster/software.htm#ctv) was employed for the heat map analysis.

### Chemicals

Sucrose, glucose, fructose, sorbitol, stachyose, and raffinose were purchased from TCI (Japan). The protein ladder (Fermentas, Canada), SYBR Premix ExTaq (TaKaRa, Bio Inc., Japan), ReverAid First-strand cDNA Synthesis Kit (Fermentas, Canada), and glutaraldehyde (TED, Pella, Inc., USA) were obtained from the indicated suppliers. All the other chemicals used were purchased from Sigma (USA).

## Supplementary Information

**Additional file 1 Fig. S1.** Anatomical characteristics of the three kinds of roots in cassava. **a** Transverse section of a primary fibrous root. **b** Transverse section of a secondary fibrous root. **c–g** transverse section of storage root in the early (**c**), middle (**d** phloem, **e** xylem) and late (**f** phloem, **g** xylem) stages. All of them were with cropped edges and adjusted position using Adobe Photoshop CS6.0 software. Bars = 1 μm.

**Additional file 2 Fig. S2.** Structure of the primary fibrous root and subcellular localization of SUTs in the developing cassava root using immunogold particle labeling. **a** SE–CC complex and its surrounding PCs, with increased intercellular space observed. **b** Plasmolysis of the CCs of a PFR, some of which exhibited plasmalemma invagination. **c** Suspected paramural bodies were observed in some CCs. **d** Amplification of the suspected paramural body. **e** Amplification of the invagination shown in **b**. **f, g** SUTs visualized with immunogold particles were localized to the plasma membranes of CCs and PCs. All of them were with cropped edges and adjusted position using Adobe Photoshop CS6.0 software.

**Additional file 3 Fig. S3.** Protein gel blot pictures of CWI, SAI and SuSy. **a** Protein gel blot of CWI. **b** Protein gel blot of SAI. **c** Protein gel blot of SuSy. **a, b** and **c** were scaned by a printer (E408, Canon, Japan) directly. **d** Protein gel blot of SuSy. **e, f** and **g** Protein gel blot of SAI, which were scanned by a printer (E408, Canon, Japan) with a blank paper as background.

## Data Availability

Raw sequence reads of the transcriptomes of the 10 samples from cassava leaves and storage roots were deposited in the NCBI Sequence Read Archive (SRA) (https://www.ncbi.nlm.nih.gov/sra?linkname = bioproject_sra_all&from_uid=248260). The amino acid sequences of CWI-1, CWI-2, CWI-5, SAI and SuSy were deposited in GenBank under the accession numbers: JQ339929, JX291160, JX201159, JN616390 and AAV74405.1, respectively. The datasets generated and/or analysed during the current study are available in the Science Data Bank repository (http://www.scidb.cn/s/p6bY3Q3), or available from the corresponding author for reasonable request.

## Abbreviations

CF: carboxyfluorescein
CC: companion cell
CFDA: nonfluorescent 6(5) carboxyfluorescein diacetate
CLSM: confocal laser scanning microscopy
CW: cell wall
CWI: cell wall acid invertase
DAPE: days after plant emergence
GFP: green fluorescent protein
HT: hexose transporter
NI: neutral invertase
SAI: soluble acid invertase
SEL: size exclusion limit
SE: sieve element
SFR: secondary fibrous root
SuSy: sucrose synthase
SUT: sucrose transporters
PC: parenchyma cell
PD: plasmodesma
PFR: primary fibrous root
PPH: primary phloem
PPU: pore-plasmodesma unit
S: starch granule
SDS-PAGE: sodium dodecyl sulfate polyacrylamide gel electrophoresis
TBS: Tris-buffered saline
TBST: Tris-buffered saline with Tween 20
TEM: transmission electron microscopy
UDPG: uridine diphosphate glucose
V: vacuole
Suc: sucrose
Glc: glucose
Fru: fructose
CATAS: Chinese Academy of Tropical Agriculture Sciences
