# Supplementary figures and images for: Predominantly symplastic phloem unloading of photosynthates maintains efficient starch accumulation in the cassava storage roots (Manihot esculenta Crantz)
Source: BMC Plant Biol. 2021 Jul 3;21:318. doi: 10.1186/s12870-021-03088-1 (PMC8254309; doi:10.1186/s12870-021-03088-1)

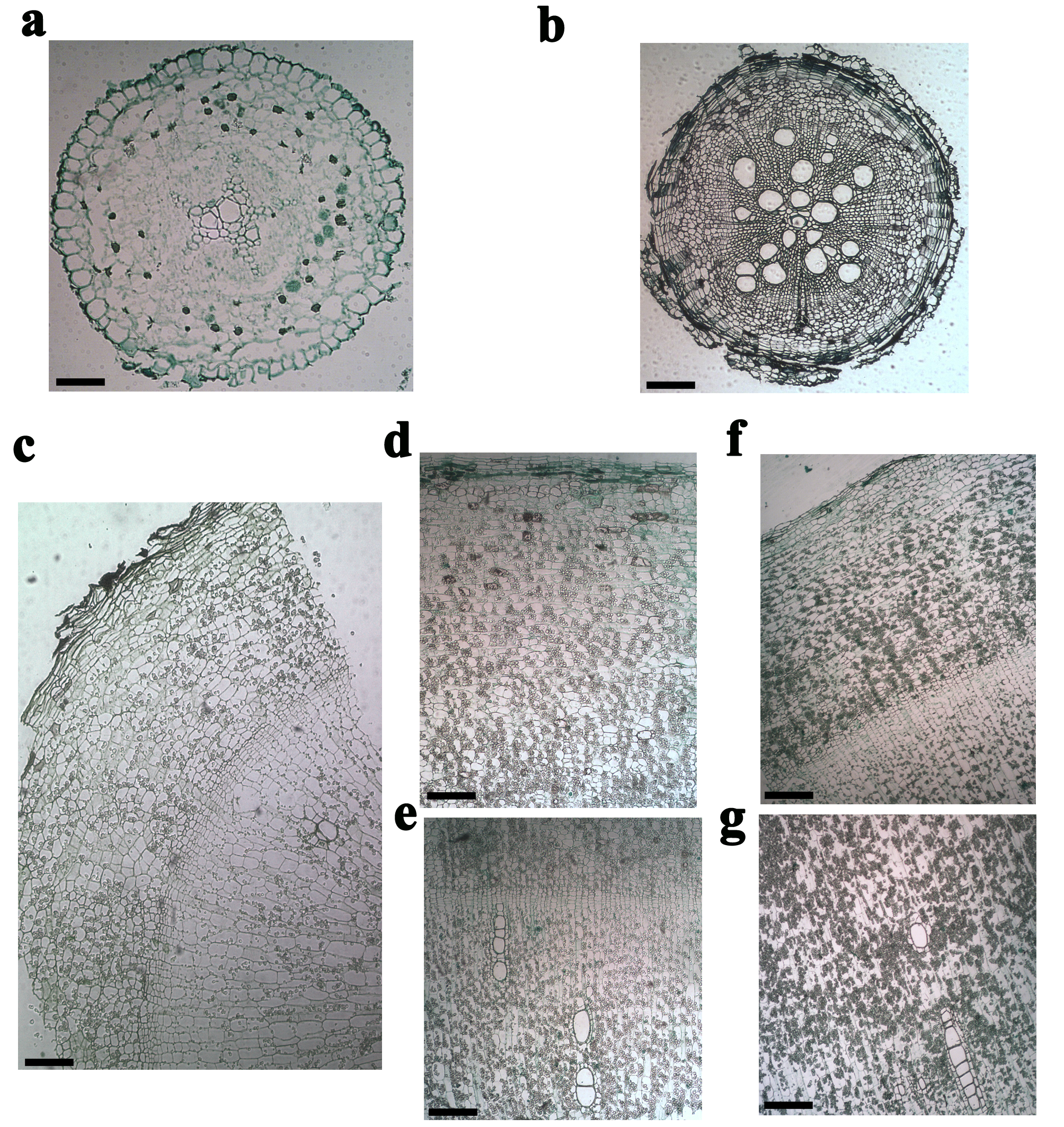

Supplement: Supplementary file 1 — Additional file 1 Fig. S1. Anatomical characteristics of the three kinds of roots in cassava. a Transverse section of a primary fibrous root. b Transverse section of a secondary fibrous root. c–g transverse section of storage root in the early (c), middle (d phloem, e xylem) and late (f phloem, g xylem) stages. All of them were with cropped edges and adjusted position using Adobe Photoshop CS6.0 software. Bars = 1 μm. [file 12870_2021_3088_MOESM1_ESM.tif]

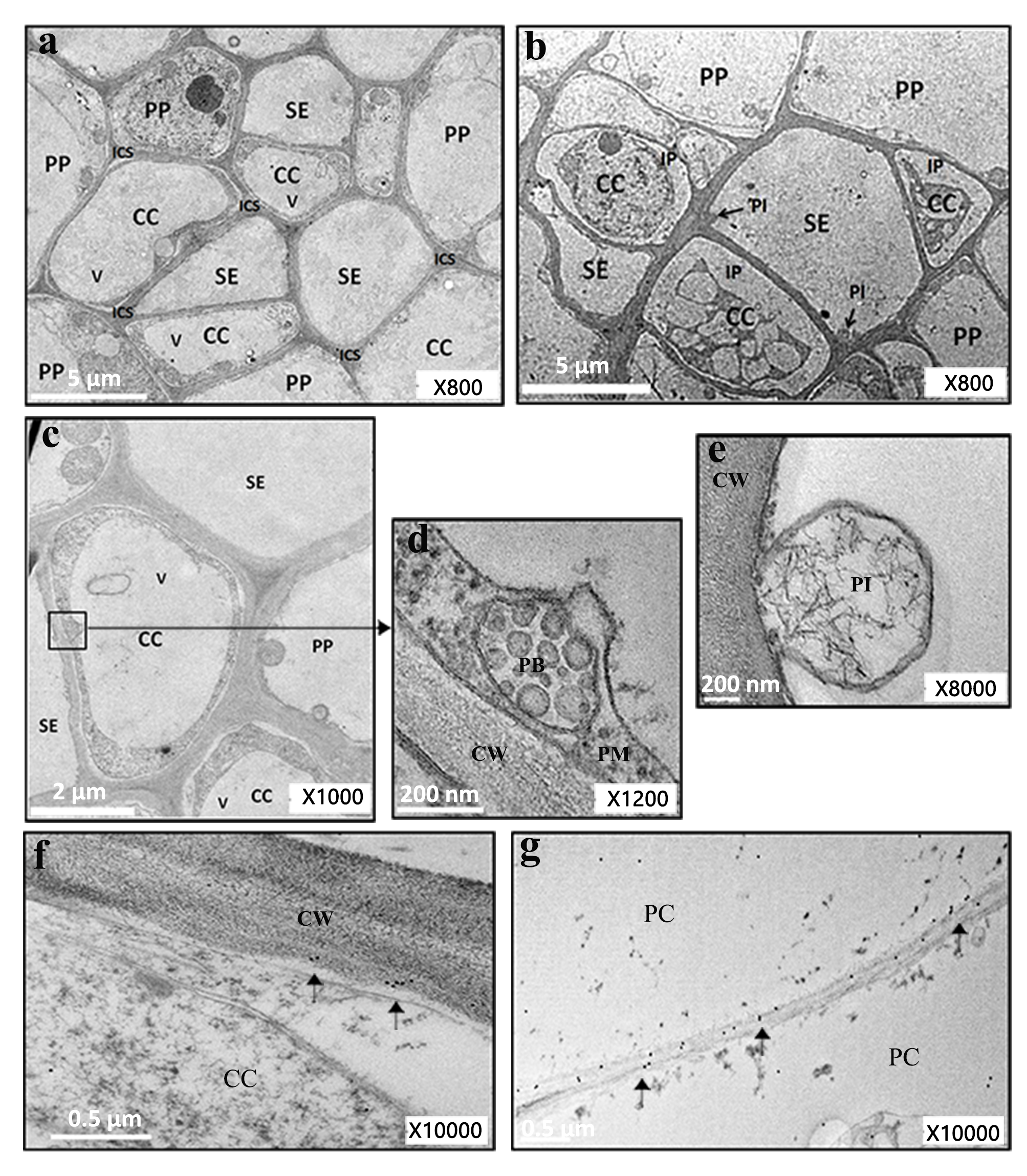

Supplement: Supplementary file 2 — Additional file 2 Fig. S2. Structure of the primary fibrous root and subcellular localization of SUTs in the developing cassava root using immunogold particle labeling. a SE–CC complex and its surrounding PCs, with increased intercellular space observed. b Plasmolysis of the CCs of a PFR, some of which exhibited plasmalemma invagination. c Suspected paramural bodies were observed in some CCs. d Amplification of the suspected paramural body. e Amplification of the invagination shown in b. f, g SUTs visualized with immunogold particles were localized to the plasma membranes of CCs and PCs. All of them were with cropped edges and adjusted position using Adobe Photoshop CS6.0 software. [file 12870_2021_3088_MOESM2_ESM.tif]

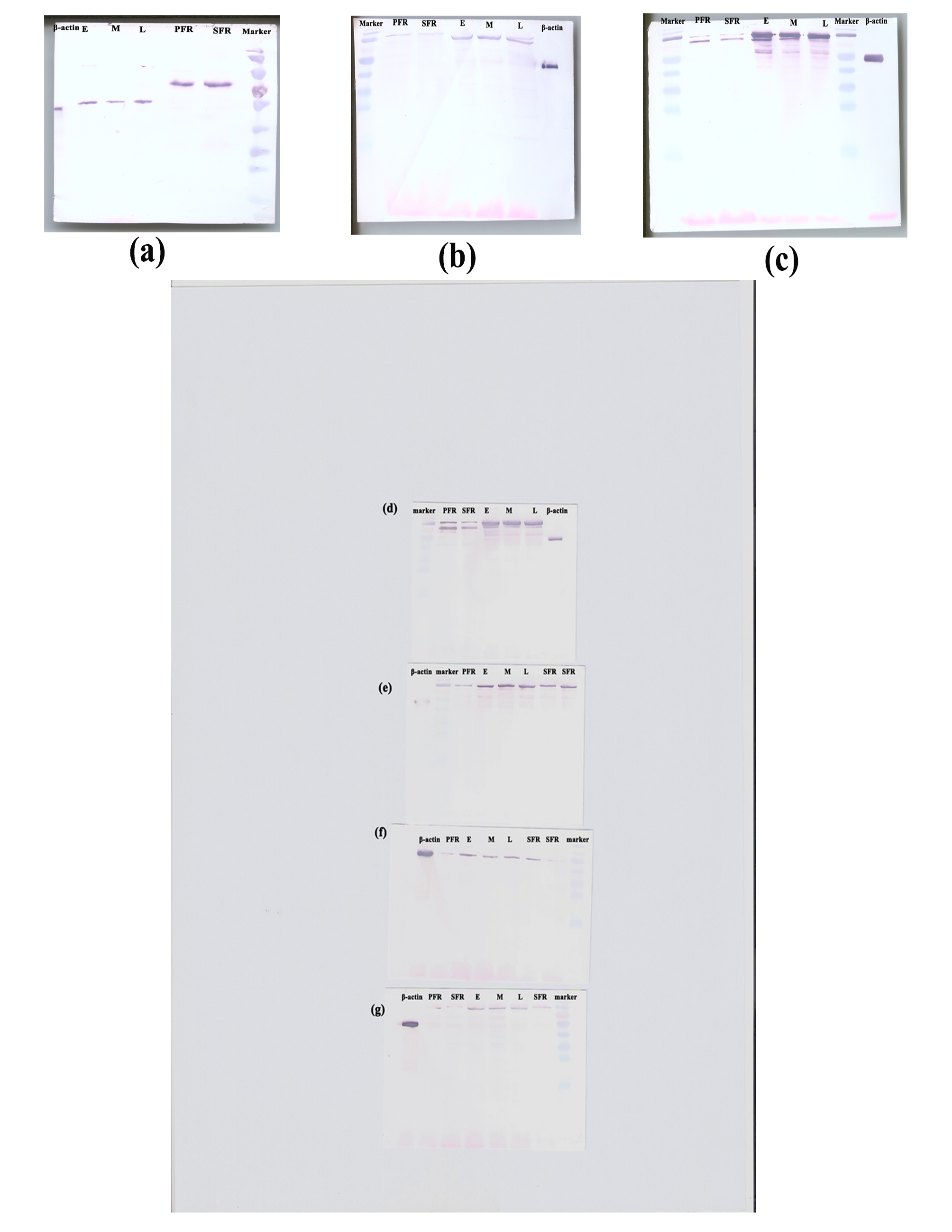

Supplement: Supplementary file 3 — Additional file 3 Fig. S3. Protein gel blot pictures of CWI, SAI and SuSy. a Protein gel blot of CWI. b Protein gel blot of SAI. c Protein gel blot of SuSy. a, b and c were scaned by a printer (E408, Canon, Japan) directly. d Protein gel blot of SuSy. e, f and g Protein gel blot of SAI, which were scanned by a printer (E408, Canon, Japan) with a blank paper as background. [file 12870_2021_3088_MOESM3_ESM.tif]
